# Cheap Polishing Wheels and Cones

**Published:** 1874-08

**Authors:** 


### CHEAP POLISHING WHEELS AND CONES.

By cutting off corn cobs various lengths, say from three-eighths of an inch to any length desired, the small end of the cob answers well for cones. You have a wheel which cuts fast and smooth, this may not be new to all but to many it is worth trying.

